# Relative age effects in German youth A and B men's soccer teams: survival of the fittest?

**DOI:** 10.3389/fspor.2024.1432605

**Published:** 2024-07-11

**Authors:** Florian Heilmann, Alexander Kuhlig, Oliver Stoll

**Affiliations:** Movement Science Lab, Institute for Sport Science, Martin-Luther University Halle-Wittenberg, Halle (Saale), Germany

**Keywords:** birth quartile, relative age effects, youth soccer, maturation, talent selection

## Abstract

The study investigates relative age effects (RAE) in German youth soccer (Youth Bundesliga A: January 2004 to December 2005 and B: January 2006 to December 2007; highest league in German youth soccer) and its persistence in third-division players. Data from the 2022–2023 season (120 teams, 3,174 players) were analyzed using chi-square tests. Significant RAE was found in the A-series (*p* < .001), B-series (*p* < .001), and third-division professionals (*p* < .001). Notably, RAE was prominent among younger players but less evident in older third-division players (*p* = .116), indicating a diminishing selection effect with age and professional tenure. Coaches and talent managers are advised to consider RAE and additional factors like player maturity in talent selection for more efficient talent management strategies, especially in youth academies.

## 1 Introduction

**Figure 1:**
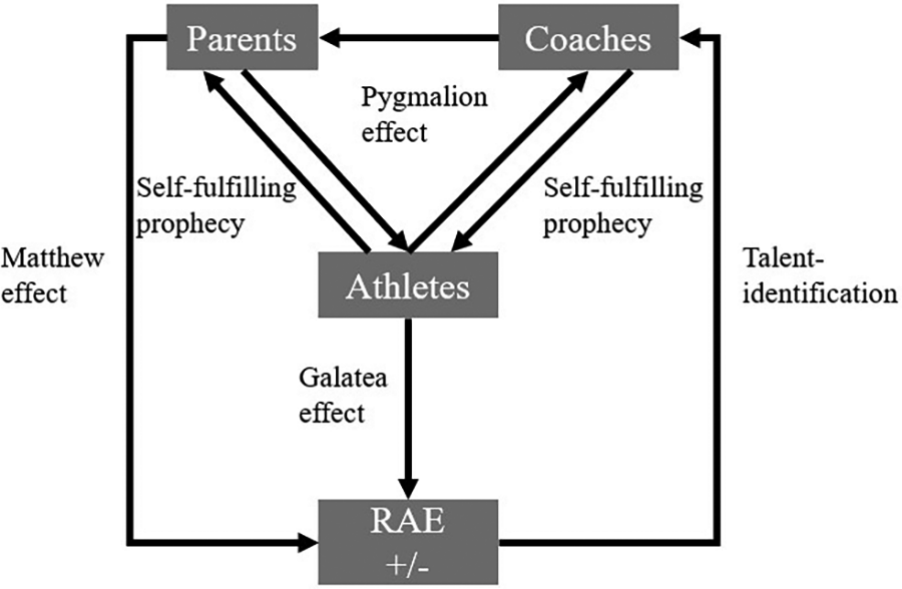
A conceptual model showing the influences on athletes' development, highlighting the Pygmalion effect, self-fulfilling prophecy, Galatea effect, Matthew effect, and talent identification. Parents and coaches shape athletes' perceptions and performance, impacting the Relative Age Effect (RAE).

In a sports context, calendar age, and birthdates are essential for grouping athletes for competitive comparison. In general, there has been a tendency to select athletes for teams born early in the year or a particular selection period (close to the cut-off date of selection). Recent studies have demonstrated that relative age effects (RAE) in sports is a global phenomenon that affects a wide range of competitive sports (1). The impact could be demonstrated for individual sports (2, 3) and team sports such as basketball (4, 5), ice hockey (6, 7) or especially for soccer (8, 9). Children and adolescents born early in the year enjoy considerable advantages over those born later in the year in terms of academic achievements (10, 11), emotional and social life (12, 13). There are various reasons for this effect and its continued existence through the older age groups in the context of sports: the Matthew effect (14), the Pygmalion effect (15), and the Galatea effect (14, 16). For example, a contrary hypothesis is postulated by Kelly et al. (17) and describes the advantages of later-born athletes (the underdog hypothesis). The Matthew effect (14), also known as the “rich get richer” effect, refers to the phenomenon where individuals or groups already successful in a particular field tend to become even more successful over time. This can happen for various reasons, such as access to resources, support, or opportunities unavailable to others. In sports, the Matthew effect can manifest as the RAE. Athletes born early are often physically more mature and developed than those born later in the year (14). As a result, they may be selected for the more advanced teams and programs and receive more coaching and training opportunities (18). Over time, this can create a self-reinforcing cycle where early-born athletes continue to dominate in their sport while late-born athletes may struggle to keep up. This can lead to a skewed distribution of success and opportunities in the sport, with the “richest” (i.e., early-born) athletes becoming even more successful. In contrast, the “poorest” (i.e., late-born) athletes fall behind (see Figure 1).

**Figure 2:**
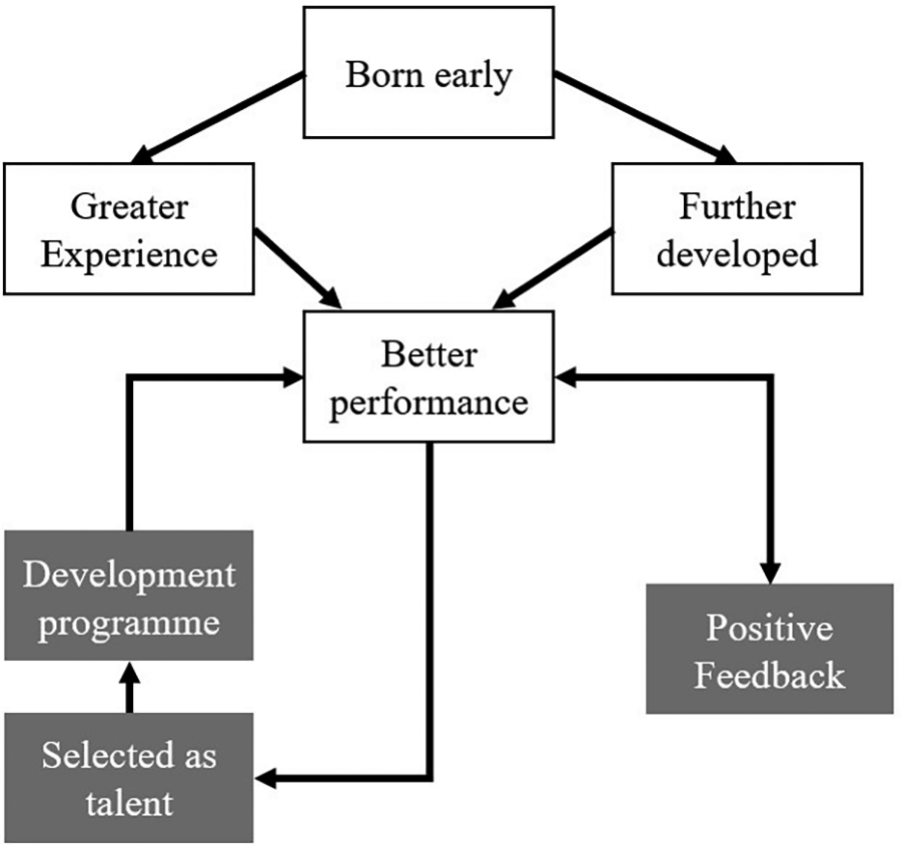
A model illustrating how being born early in the selection period can lead to greater experience and further development, resulting in better performance. This improved performance leads to positive feedback and selection into development programs, reinforcing the athlete's recognition as talent.

The Pygmalion effect (15), also known as the Rosenthal effect, refers to the phenomenon in which higher expectations of individuals lead to increased performance. With the RAE, athletes born early in the year are more likely to be selected for professional teams and programs (i.e., youth academies). This results in higher expectations of the coaches and scouts. As a result, the abovementioned Matthew effect is supported. This can contribute to a self-fulfilling prophecy, as the increased expectations lead to improved performance, leading to even higher expectations. The Galatea effect (14, 16) is a phenomenon where individuals who are given positive feedback and encouragement tend to perform better than those who do not receive such feedback. In the context of RAE, early-born athletes are more likely to receive positive feedback and encouragement from coaches and surroundings due to the abovementioned advantages. This could again lead to a self-fulfilling prophecy, where these athletes continue to receive positive feedback and encouragement, leading to better performance and more opportunities to advance in their sport. As a result of RAE, the late-born players may be labeled as “underdogs” in the context of the underdog hypothesis (17). They may have to work harder to prove themselves and earn opportunities to advance in their soccer careers (see Figure 2). According to review studies and meta-analyses, numerous factors moderate the RAE in sport (19, 20). These include playing position, gender, age, level of competition, and setting of the sport.

In professional soccer, the RAE significantly impacts the talent selection and development process. For example, a multi-country study by (21) found an over-representation of players born in the first birth quartile (BQ) in national and professional youth selections across all age groups. The statements apply primarily to male footballers, because for female players the development stage and the composition of the squad still play a key role. Massa et al. (22) discovered a comparable effect in professional football in Brazil. As evidenced by the strong RAE in youth football that has been found in America, Australia, Brazil, Germany, and Japan, among other places, there may be a consistent worldwide effect at play here that is not influenced by national variations in the dates used to determine the start and end of the sporting year (23). A remarkable reduction in RAE effects (24) has not been achieved in the last decades. Recent research shows that maturation has got a higher impact on performance than RAE (25). Nevertheless, Hill et al. (26) argue that RAE and maturation are independent constructs. Thus, different strategies to prevent RAE and selection bias regarding the maturation of players are needed (20, 27). Coaches and scouts should consider RAE in their decisions. RAE could cause individual differences in up to one year.

In German soccer, the findings of Votteler et al. (28) reveal significant direct and indirect RAEs for physiologically demanding tests and almost no effects for technically demanding tests. The study of Helsen et al. (29) shows that relative age effects exist in German youth soccer. However, no advantage in anthropometric or performance-related characteristics can account for it. A player's chances of becoming a professional later in their career are higher for younger players chosen for national teams (30). Götze et al. (31) could identify the influence of gender and competition level on the RAE in German soccer. Their data indicates a RAE in German adult soccer for both males and females, which may be coupled with a loss of great players who were once highly valued during their childhood years. As a result, fewer skilled players would be available for the adult division. The effect sizes for the RAE are large in the U19 and small to medium from U20 to the first league (including the national team and first and second Bundesliga). The German youth soccer system (before 2023), particularly through the B-Junioren Bundesliga (U17) and A-Junioren Bundesliga (U19), provides a structured and competitive environment for young talents. These leagues are pivotal in the development of players, many of whom progress to professional careers in Germany's top leagues or internationally. The system is supported by rigorous scouting, professional club academies, and a clear pathway from youth to senior professional soccer. The pressure on the youth soccer players, especially for academy players is very high. There are high expectations, intense competitions and they have to balance education and soccer. In this case, it is important to examine single soccer nations, because they potentially differ from others and it allows to compare the different pathways and contextual factors of RAE.

The position on the field plays an important role when analyzing the RAE in athletes (32–34). For example, Schorer et al. (34) could show no significant RAE for circle players and goalkeepers in handball. Professional U-20 South American soccer players were the subject of an investigation by (35), who noted RAE at all positions played except for goalkeepers. Hurley and colleagues (2019) could not find RAE for forwards and goaltenders. There is evidence that goaltenders generally do not show significant RAE. Schorer et al. (34) explain this with different demands for these playing positions, especially for goaltenders. Further studies have indicated that central defenders and midfielders have indicated greater RAE prevalence compared to other positions on the field (36) (Finnegan et al., 2024).

Doyle et al. (18) analyzed the data of the 1,000 best professional footballers (by market value on transfermarkt.de) and found that these players were born earlier than could be expected by chance. The level of competition seems to be a moderation factor for RAE. Furthermore, admission to youth academies plays an essential role in the occurrence of RAE (37). The study of Grossmann and Lames (37) shows that a strength RAE is even more present in youth academy players than in amateur U17 and U19 players. There is significantly less evidence concerning the longitudinal progression of RAE in youth soccer. The only research that indicates a longitudinal analysis of talent selection processes reveals an increase in RAE for players who are newly selected for higher competition levels and no change in RAE extent for players who are retained at the same competition level across successive age categories is the work by Votteler and Höner (23). According to Cobley et al. (1) and Szwarc et al. (38), it is plausible that the elimination of disparities in physical maturity is a contributing factor, meaning that athletes who are relatively younger no longer face any disadvantages (underdog hypothesis). According to Cobley et al. (1), elder athletes switching to different sports may also contribute. Due to overtraining, burnout, or other issues, older athletes who trained hard to reach a high-performance level in their junior years may also decide not to compete in sports. Evidence suggests that specialized training environments are associated with shorter playing careers and higher adult dropout rates in sports, including ice hockey and soccer (1). Moreover, the studies of Dugdale et al. (39) show that RAE does not always translate into senior or adult level, and Andrew et al. (40) show that it does not always lead to success.

Nevertheless, the current findings regarding the RAE often neglect the effect of the level of competition and the transition between age groups, youth academies, and professional soccer leagues. Furthermore, the research often lacks information on the prevalence of RAE in different regions of the examined countries. These findings could help to find sufficient strategies to prevent RAE selection bias. The present study aimed to describe the prevalence of RAE in German soccer and explain it by different explanatory variables such as age group, playing position, performance in the league (by top clubs) and the region of the country.

## 2 Methods

### 2.1 Participants

The players of the German Youth Bundesliga A (January 2004 to December 2005) and B (January 2006 to December 2007; the highest league) and the players in the 3. Liga in Germany (third division) were analyzed in this study. One hundred twenty teams and a total of 3,174 players were included (extracted from valid database transfermarkt.de). The twelve months of the year were divided into four birth quartiles (BQs). January, February, and March were classified as “BQ1”, April, May and June were classified as “BQ2”, July, August and September were classified as “BQ3”, and October, November, and December as “BQ4”. Birth quartiles were compared with the general population data from 2010 because there were no detailed birth distribution datasets from other years. The distributions did not significantly differ compared with the 2010 data (statista.de). An overview of the characteristics of the three series (Youth A and B Bundesliga and third division in Germany) is displayed in Table 1. The cut-off date for the German youth soccer leagues is the 1st of January. Furthermore, the region (north/north-east, west, south/south-west), the position on the field [goaltender (1), defender (2–6), midfielder (8), offender (7, 9, 10, 11)], and the top teams (top 5 and last 5) were analyzed as covariates. The database often not identify the exact position on the field but one of the above-mentioned category.

**Table 1:** Composition of the sample by leagues (U17/19) in season 2022/2023.

Age group	North/north-east	West	South/south-west	Total (*n*)
U17	394	389	391	1,174
U19	443	424	480	1,347
3. division				571
Total (*n*)				3,092

### 2.2 Statistical analysis

The data were first checked for plausibility. No outliers had to be excluded, but for a total of 36 players, an exact birth date could not be evaluated. Chi-square (*χ*2) analysis was used to compare quartile distributions in the sample and against population values, following procedures outlined by Kelly et al. (17). This test does not reveal the magnitude of difference between quartile distributions for significant chi-square outputs, so Cramer's *V* was used. According to accepted correlation standards, the Cramer's V was read as follows: a value of 0.06 or higher would suggest a small impact size, a value of 0.17 or higher would indicate a medium effect size, and a value of 0.29 or higher would indicate a big effect size (41). Since the results are always undirected due to the squaring in chi-square analyses, a subsequent graphical inspection of the data is necessary to make substantive statements about the RAE in the sample.

## 3 Results

Table 2 shows that the distribution of the month of birth differed significantly (*p* < .05) from the distribution of births in Germany in 2010 in all scales of the U17. A significant effect size (*V* ≥ 0.30) and, thus, a large RAE could be demonstrated in all regions.

**Table 2:** Distribution of birth quartiles (%) in the analyzed age groups in the 2022/2023 season compared to the German birth statistics from 2010 (available, comparable age group).

Age group	Classification	*n*	BQ1 (%)	BQ2 (%)	BQ3 (%)	BQ4 (%)	*χ* ^2^	*p*	*V*	Effect
U17	North/north-east	394	42.12	25.89	24.11	7.87	103.20	<.001	0.30	Large
	West	389	42.16	31.11	15.17	11.57	110.22	<.001	0.31	Large
	South/south-west	391	47.57	25.06	18.67	8.70	144.39	<.001	0.35	Large
	Young (2007)	100	52.00	21.00	14.00	13.00	45.93	<.001	0.39	Large
	Old (2006)	1068	43.16	28.00	19.94	8.90	303.71	<.001	0.31	Large
	Total	1,174	43.95	27.34	19.34	9.37	344.01	<.001	0.31	Large
U19	North/noth-east	443	38.37	30.47	16.25	14.90	82.96	<.001	0.25	Interm
	West	424	40.33	29.95	19.34	10.38	99.17	<.001	0.28	Interm
	South/south-west	480	42.92	26.67	17.29	13.13	118.33	<.001	0.29	Interm
	Young (2,005)	682	41.94	29.33	17.60	11.14	175.27	<.001	0.29	Interm
	Old (2004)	649	39.14	28.20	17.87	14.79	114.68	<.001	0.24	Interm
	Total	1,347	40.61	28.95	17.59	12.84	294	<.001	0.27	Interm
third division	Young (born after 1998)	307	32.57	28.01	26.06	13.36	28.05	<.001	0.17	Small
	Old (born before 1998)	264	29.55	24.24	25.76	20.45	5.91	.116	0.09	Small
	Total	571	31.17	26.27	25.92	16.64	29.69	<.001	0.13	Small
German birth statistics (2010)		677,947	23.76	24.43	26.98	24.84				

**Figure 3:**
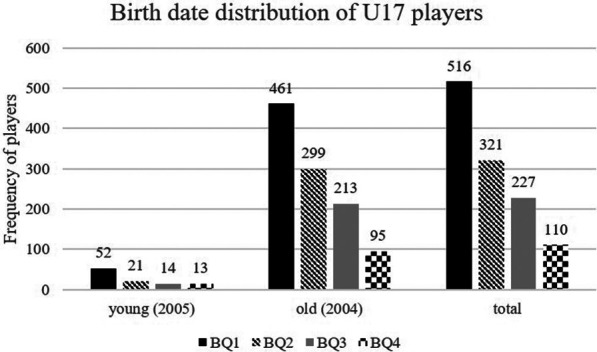
Birth date distribution of U17 players (Youth B).

The most substantial effect was found in the south/south-west region (*χ*^2^ = 144.39, *p *< .001, *V* = 0.35), in which around 73% of players were born in the first half of the year (BQ1 and BQ2). When investigating the differences between the two age groups in the U17, players born in 2007 (*χ*^2^ = 45.92, *p* < .001, *V* = 0.39) showed a higher effect size for RAE than players born in 2006 (*χ*^2^ = 303.71, *p* < .001, *V* = 0.31). The absolute number of players per quartile decreases significantly with decreasing age. While most players were born in Q1 of 2006 (*n* = 461), Q4 of 2007 had the fewest players in the U17 teams (*n *= 13; see Figure 3). Overall (*n *= 1,174), the players in the U17 national league shows a large effect size (*χ*^2^ = 344.01, *p* < .001, *V* = 0.31) concerning the differences in the distribution of birth quartiles to the reference population (RAE). Around 71% of all U17 Bundesliga players were born in the first half of the year (BQ1 and BQ2).

**Figure 4:**
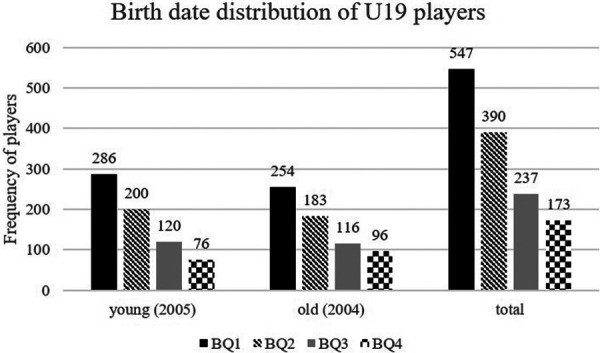
Birth date distribution of U19 players (Youth A).

The effect sizes of the chi-square test in the U19 age group were not as large as in the U17 age group. Nevertheless, RAEs were also found in this age group. All squadrons showed a significantly different distribution from the reference population and a medium effect size. The south/south-west region shows the strongest effect size for RAE (*χ*^2^ = 118.33, *p* < .001, *V* = 0.29). In the individual age groups, the 2005 players (*χ*^2^ = 175.27, *p* < .001, *V* = 0.29) had a higher RAE than the 2004 players (*χ*^2^ = 114.68, *p* < .001, *V* = 0.24). In the U19, most players were born in BQ1 and the fewest in BQ4 in both birth years (2004: *n* = 649; 2005: *n* = 682). There is a consistent drop in players from BQ1 to BQ4 (see Figure 4). As a result, from BQ1 to BQ4 of 2004, the number of players decreases steadily, then increases in Q1 of 2005 before declining again until Q4 of 2005. For all U19 national leagues combined, a medium effect size was seen for the RAE (*χ*^2^ = 294, *p* < .001, *V* = 0.27). 69.56% of the players were born in the first half of the year (BQ1 and BQ2). For all U19 national leagues combined, a medium effect size was seen for the RAE (*χ*^2^ = 294, *p *< .001, *V* = 0.27). A significant RAE could also be demonstrated for the professional sector in the 3rd division. However, the effect size was smaller (*χ*^2^ = 29.69, *p* < .001, *V* = 0.13) than for the U17 and U19. Overall, 57.44% of all players in the 3rd division were born in the first half of the year (BQ1 and BQ2), which is closer to the reference population compared to the juniors, in which 71.29% (U17) and 69.56% (U19) were born in the first half of the year. Within the 3rd division, only players born after 1998 showed an RAE (*χ*^2^ = 28.05, *p* < .001, *V* = 0.17). For players born before 1998, the deviation of the birth data from the reference population was insignificant (*p* = .116). The 3rd division group (*n *= 571) also included some young players registered for the U19 junior team of the respective club in the U19 Bundesliga (*n *= 14). These players were included in the data analysis for both the juniors and the 3rd division.

**Figure 5:**
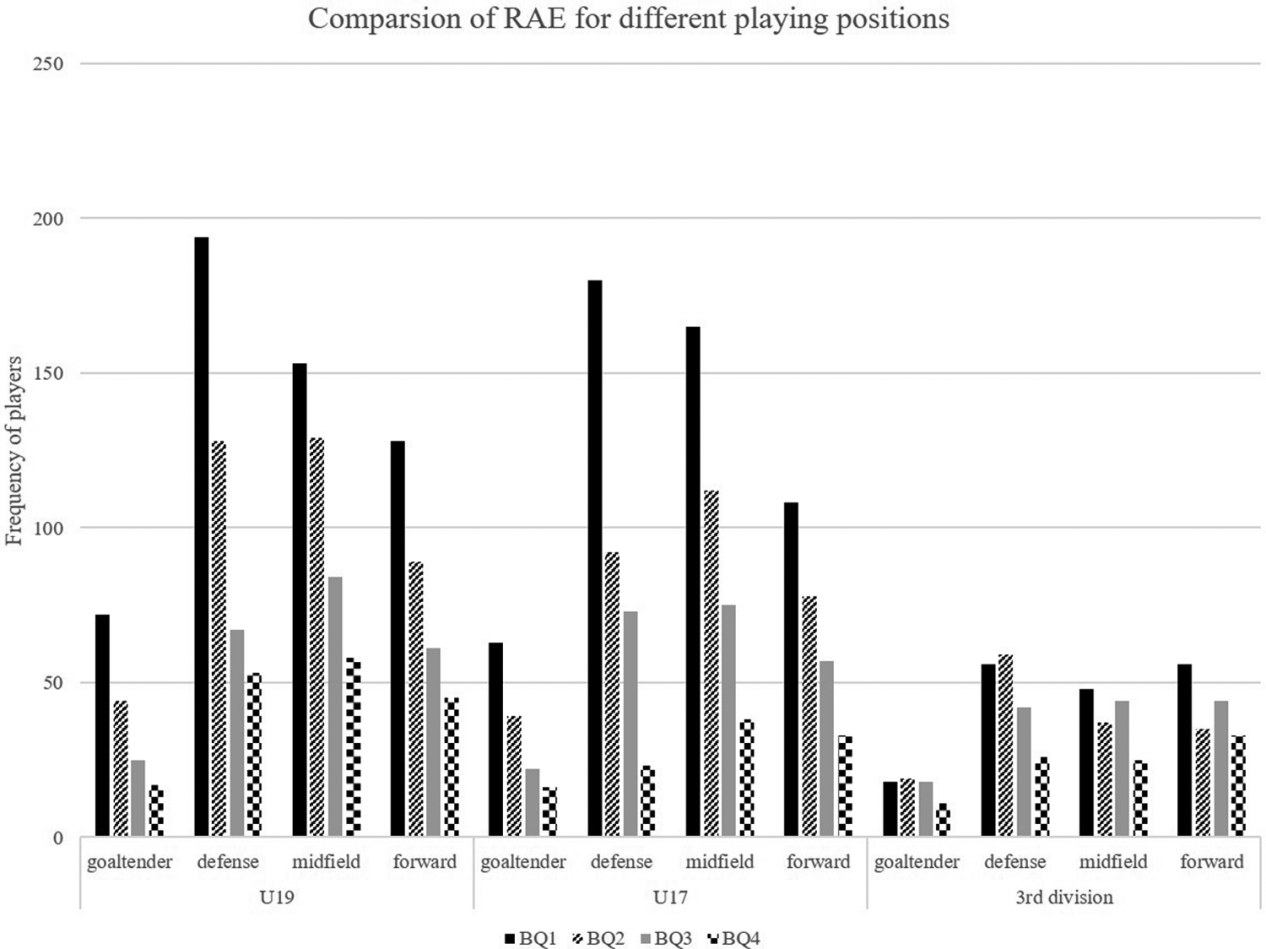
Comparison of RAE for different playing positions on the field of U19 (Youth A), U17 (Youth B) and third division.

The distribution of birth quartiles, considering the position on the field, shows larger effect sizes for goaltenders and defenders compared with midfielders and offenders (see Supplementary Table S3 and Figure 5). In theory, significant RAEs were discovered for every position on the field. Regarding the distribution's deviation, only the third division's goalkeeper position group (*χ*^2^ = 2.63, *p* = .45, *V* = 0.12) shows no significant RAE.

The results of the deviation in the birth distribution based on performance in the respective league are displayed in Supplementary Table S4. Generally speaking, every group shows a modest RAE; however, the last five teams in the third league table show non-significant chi-square tests for RAE (*χ*^2^ = 2.81, *p* = .422, *V* = 0.08). The effect size is always larger for teams ranked among the top teams in the leagues. The effect size decreases with increasing age.

In general, all age groups showed relative age effects. A consistent gradient in the distribution of the quartiles in the birth dates (BQ1 to BQ4) for all age groups is also revealed by the graphical inspection, as shown in Figure 5.

The ratios of BQ2 and BQ3 were nearly identical in the third division. Most players across all age categories were born in Q1, the fewest in Q4, and the third division had the highest percentages in Q3 and Q4. When comparing the age groups, there is a decline in the RAE as age increases. The U17 national leagues exhibited the highest RAE, and the third league displayed a comparatively smaller RAE.

## 4 Discussion

The study aimed to analyze relative age effects among German juniors A and B as well as the players of the third division (German Bundesliga and 3. Liga) in the 2022/2023 season. Therefore, the 2,521 birth dates of junior players and 571 birthdates of players in the 3rd division were analyzed. The 3,092 birth dates were divided into four quartiles, and the birth date distributions were compared with Germany's birth statistics from 2010. Furthermore, the effects of position on the field and the rank of the associated team in season 2022/23 were calculated. Generally, it could be shown that an RAE exists in every age group despite the older players of the third division.

The findings are in line with the general evidence that players born in the first birth quarter (BQ) are overrepresented in professional youth soccer across different age groups (21–23). Only the older players competing in the third division of the German Bundesliga do not show a significant deviation compared with the average population. This aspect could underpin the “underdog hypothesis”. It could be speculated that the players reaching older ages in the professional league had to overcome challenges arising from the initial disadvantages of being born late in the year. The non-translation in senior soccer leagues is in line with the findings, for example, of Andrew et al. (40) and Dugdale et al. (39). The hypothesis would claim that these players worked harder, and because of that, they could stay at this level. Götze and Hoppe (31) also show the influence of the competition level on the RAE in German professional soccer. The current findings show the same phenomenon. The RAE decreases with the age group and the competition level [i.e., U19 > U21 > first division; (31)]. The results of Doyle and Bottomley (18), which state that players with a higher value were born earlier than could be expected by chance, could not be confirmed or disproven because the younger players in our study do not have a value on the market and it was not the aim of the current study. The results of Votteler and Höner (23) longitudinal analysis of talent selection procedures, which indicate that players newly selected for higher competition levels have a higher RAE and that players retained across consecutive age categories at the same competition level do not have a different RAE, are not consistent with the current findings.

As already described, no data was collected for this study that would allow measurable conclusions to be drawn about the causes of RAEs based on the models presented by Hancock et al. (16) and Wattie et al. (42). We can only make assumptions in this regard. It is possible that relatively older people experienced advantages within the system of social actors through Matthew effects, Pygmalion effects or Galatea effects (6), entered the “vicious circle” of the dynamic model through an initial advantage, with two self-reinforcing processes increasing their lead, or were best adapted to the interactions of their constraints with environmental and task-related constraints in the constraint-based model (42), resulting in their overrepresentation in the sample.

Given their positions on the field, goaltenders and defenders have bigger effect sizes in the birth quartile distribution (RAE) than midfielders and offenders. Nevertheless, significant RAEs were discovered for every field position except for the third division's goalkeeper position group. Our results align with the findings of Campos et al. (35) regarding the lack of RAE for goaltenders in the professional league examined in the study (third division). Still, the current results show a significant RAE for younger goaltenders (U19 and U17). It seems that the distribution is oriented on the overall distribution of RAE (U17 < U19 < third division) also for goaltenders. It is plausible that the different demands on goalkeepers lead to the difference in RAE. The “underdog hypothesis” can be cited as an explanation for the generally decreasing relevance of the RAE with the age of the athletes in the current study.

Nevertheless, the level of competition seems to be a moderation factor for RAE. Each group generally displays a moderate RAE, yet the last five clubs in the third league table see non-significant chi-square tests for RAE (*χ*^2^ = 2.81, *p* = .422, *V* = 0.08). When a team is ranked among the best teams in the league, the effect size for RAE is always bigger. The findings align with Grossmann and Lames (37) and Augste and Lames (8). They could show significant correlations between the final rank of the teams and the median. Thus, the older the team is (BQ1 < BQ4), the better the rank. We could not explain the differences in effect sizes between the different regions (north/north- east; west; south/south-west) because the compilation of the clubs leaves no room for speculation regarding performance of the different clubs and the prevalance of RAE. The only plausible reason for the higher effect sizes in the south/south-west group could be the high population numbers in these regions, which can lead to a high selection pressure overall.

Several limitations should be considered when interpreting the results of this study. First, the division of birth dates into quartiles is associated with a certain degree of arbitrariness, and it could result in a difference of one month (or even one day) having a more significant effect in one case and no effect at all in another, such as March 31 compared to January 31. Furthermore, the quartiles may contain different numbers of days, which can affect comparability (42). Secondly, the date of birth alone cannot reliably determine how mature a player is compared to other players his age. However, it was impossible to consider player maturity in the study. Only year of birth, birth quartile, position, and team ranking could be analyzed. Thirdly, in this study, the A and B junior national leagues were examined for the occurrence of an RAE. In addition, the birth dates of the 3rd division were analyzed because it was assumed that a certain number of players in the A and B junior national leagues initially switched to the 3rd division. It is also possible that many players trained at the youth academy and in the youth leagues move directly to the first or second division (German Bundesliga). However, the statistics, which show a sharp decline in the use of domestic U21 players in the first division (German Bundesliga) between 2017/2018 and 2019/2020, tend to suggest the opposite. A certain degree of inaccuracy must also be assumed concerning the data collected. The playing positions listed on the internet may not always correspond to reality. Positions are often swapped or changed for training, particularly at the junior level. In addition, players can interpret soccer positions differently, making distinguishing between offensive and defensive positions difficult. They may also make inaccurate assumptions about the cause of the birth distribution for certain positions. Furthermore, only the detailed birth distribution of 2010 was available because this was a year of the census.

The current study shows an RAE for the highest German youth league could be identified. Furthermore, the youngest players are born earlier in the year than the older players in the third division of the German Bundesliga. Although, in contrast to maturity, relative age appears to be far less significant for physical performance in most cases (27), the selection process in the early years of talent development could impact the athletes' careers. At a high selection level, however, the realization remains that even relatively younger players show particularly high performance in motor performance diagnostics as well as for game intelligence, tactical, and psychological skills (Williams et al. (43) despite their age-related disadvantages (28). This makes their motor skills all the more important than those of relatively older players, which they must demonstrate to be accepted into the DFB talent development program (23). They must develop special physical, tactical and technical skills to be competitive (24). The problem is that young players who are not as physically developed could give up due to the constant disadvantage, which leads to high dropout rates in adolescence (1, 30).

Further research should focus on measures to reduce the RAE. For example, Sierra-Diaz et al. (20) recommend different strategies, such as altering or rotating the annual cut-off date, to create alternative ways to group athletes for competition (i.e., anthropometric attributes) or to develop internal reforms in soccer academies and enhance some competitive regulations. Further strategies could be to assess the biological maturity or stage of development oft he players.

A follow-up study seems rewarding because the German Football Federation revised the competition mode for the highest youth league (U19 and U17). The A and B Junior Bundesliga have been divided into three leagues. At the start of the 2024/2025 season, the U19 and U17 DFB Junior League will replace this tier system. There will be two phases: first, a regional preliminary round, and then, a main round in the second half of the season, which will be divided into League A and League B. The German champion is determined from the teams that qualify for League A. In the U 19 and U 17 DFB Junior League, Bundesliga and amateur clubs play in the same league from the outset. All clubs with a performance center are permanently qualified for the DFB Junior League. This change in competition mode could reduce RAE selection bias because the coaches could use their players more independently of their current performance and substitute seven players per game in the new mode. The follow-up study could show if the prevalence of RAE changes over time. Nevertheless, the decisive role of the coaches must be mentioned at this point. They have to be aware of the prevalance of RAE and have to take the findings into account in order to select or develop talents in German soccer.

## Data Availability

The original contributions presented in the study are included in the article/Supplementary Material, further inquiries can be directed to the corresponding author.
