# Supplementary material for: Relative age effects in German youth A and B men's soccer teams: survival of the fittest?
Source: Front Sports Act Living. 2024 Jul 11;6:1432605. doi: 10.3389/fspor.2024.1432605 (PMC11269141; doi:10.3389/fspor.2024.1432605)
Supplement: Supplementary file 1 [file Table3.docx]

**Supplementary Table 3.** Distribution of players across various positions (goaltender, defense, midfielder, offense) within three categories: U19, U17, and 3rd division. The absolute numbers and percentages represent players in each positional group for the four quartiles (BQ1, BQ2, BQ3, BQ4).

|  |  | **BQ1** |  | **BQ2** |  | **BQ3** |  | **BQ4** |  | **x2** | **Cramers V** | **effect size** |
| --- | --- | --- | --- | --- | --- | --- | --- | --- | --- | --- | --- | --- |
|  |  | **absolute** | **%** | **absolute** | **%** | **absolute** | **%** | **absolute** | **%** |  |  |  |
| **U19** | **goaltender** | 72 | 45,57 | 44 | 27,85 | 25 | 15,82 | 17 | 10,76 | x2(3,157) = 52,28, p <.001 | 0,33 | large |
|  | **defense** | 194 | 43,89 | 128 | 28,96 | 67 | 15,16 | 53 | 11,99 | x2(3,441) = 131,37 p <.001 | 0,31 | large |
|  | **midfielder** | 153 | 36,08 | 129 | 30,42 | 84 | 19,81 | 58 | 13,68 | x2(3,423) = 62,68, p <.001 | 0,22 | intermediate |
|  | **offense** | 128 | 39,63 | 89 | 27,55 | 61 | 18,89 | 45 | 13,93 | x2(3,322) = 58,83, p <.001 | 0,25 | intermediate |
| **U17** | **goaltender** | 63 | 45,00 | 39 | 27,86 | 22 | 15,71 | 16 | 11,43 | x2(3,139) = 43,98, p <.001 | 0,32 | large |
|  | **defense** | 180 | 48,91 | 92 | 25,00 | 73 | 19,84 | 23 | 6,25 | x2(3,367) = 156,19, p <.001 | 0,38 | large |
|  | **midfielder** | 165 | 42,31 | 112 | 28,72 | 75 | 19,23 | 38 | 9,74 | x2(3,389) = 103,86, p <.001 | 0,30 | large |
|  | **offense** | 108 | 39,13 | 78 | 28,26 | 57 | 20,65 | 33 | 11,96 | x2(3,275) = 51,64, p <.001 | 0,25 | intermediate |
| **3rd division** | **goaltender** | 18 | 27,27 | 19 | 28,79 | 18 | 27,27 | 11 | 16,67 | x2(3,65) = 2,63, p = .45 | 0,12 | small |
|  | **defense** | 56 | 30,60 | 59 | 32,24 | 42 | 22,95 | 26 | 14,21 | x2(3,182) = 17,60, p <.001 | 0,18 | intermediate |
|  | **midfielder** | 48 | 31,17 | 37 | 24,03 | 44 | 28,57 | 25 | 16,23 | x2(3,153) = 8,30, p <.05 | 0,13 | small |
|  | **offense** | 56 | 33,33 | 35 | 20,83 | 44 | 26,19 | 33 | 19,64 | x2(3,167) = 9,234, p <.05 | 0,14 | small |
