# Supplementary material for: Relative age effects in German youth A and B men's soccer teams: survival of the fittest?
Source: Front Sports Act Living. 2024 Jul 11;6:1432605. doi: 10.3389/fspor.2024.1432605 (PMC11269141; doi:10.3389/fspor.2024.1432605)
Supplement: Supplementary file 2 [file Table4.docx]

**Supplementary Table 4.** Distribution of players in the best and last five performance categories across U19, U17, and 3rd division levels. The absolute numbers and percentages represent players within each performance category for the four quartiles (BQ1, BQ2, BQ3, BQ4).

|  |  | **BQ1** |  | **BQ2** |  | **BQ3** |  | **BQ4** |  | **chi square test** | **Cramers V** | **effect size** |
| --- | --- | --- | --- | --- | --- | --- | --- | --- | --- | --- | --- | --- |
|  |  | **absolute** | **%** | **absolute** | **%** | **absolute** | **%** | **absolute** | **%** |  |  |  |
| U19 | **best 5** | 181 | 45,36 | 103 | 25,81 | 67 | 16,79 | 48 | 12,03 | x2(3,398) = 120,4, p <.001 | 0,32 | large |
|  | **last 5** | 148 | 36,82 | 111 | 27,61 | 87 | 21,64 | 56 | 13,93 | x2(3,401) = 54,01, p <.001 | 0,21 | intermediate |
| U17 | **best 5** | 188 | 51,79 | 93 | 25,62 | 54 | 14,88 | 28 | 7,71 | x2(3,362) = 182,82, p <.001 | 0,41 | large |
|  | **last 5** | 147 | 42,36 | 94 | 27,09 | 74 | 21,33 | 32 | 9,22 | x2(3,346) = 89,73, p <.001 | 0,29 | intermediate |
| 3rd division | **best 5** | 43 | 31,16 | 44 | 31,88 | 35 | 25,36 | 16 | 11,59 | x2(3,137) = 16,2, p <.01 | 0,20 | intermediate |
|  | **last 5** | 38 | 26,95 | 38 | 26,95 | 38 | 26,95 | 27 | 19,15 | x2(3,140) = 2,81, p = .422 | 0,08 | small |
